# Supplementary material for: Chromothripsis during telomere crisis is independent of NHEJ, and consistent with a replicative origin
Source: Genome Res. 2019 May;29(5):737–49. doi: 10.1101/gr.240705.118 (PMC6499312; doi:10.1101/gr.240705.118)
Supplement: Supplemental Material [file supp_gr.240705.118_Supplemental_file_1.zip › contigs/annotated_contigs/DB57/contig.2.DB57_length_492_mean_cov_6.9918699187.docx]

**DB57_length_492_mean_cov_6.9918699187**

```
AATTGTACATAGGAGCGGCTACTCACAC|GCACCCATCAAGAAAAGTTACTCAAAATGGAAACGGGTCTGCAGTCACCACTGATGAGTC
                             >chr18:33935509-33935809 - E=5e-169
ACTTGCATCTCCCTCGTGTGTGATTGAGAATTAAGAGAAGGTCAAAGCCATGGTAGTTCCTCCTAAATACTTTAAGGCCTCAATGTACC

CCCATTTCCTGGTAGGTGGGGACCAGTCAGCAGGGTGAACAGTTGCTCTTACCCGGCGTCATCCTGGAAGCCTTCCAACTCATCCCGCT

GCTCTGCCAGGGTGGCCTCCAAATCCAGGTAGGCGCCATTGTGAGAGAGCTGCAGAGTACAG|CTGTCGCTATGCTGGAGTGCAGTGGT
                                                                >chr18:34121032-34121196
GCGATCTTGGCTCACTGCAACCTCCACCTCCCGGGTTCAAGTGATTCTCCTTCCTCAGCCTCCGGAGTAGCTGCGATTACAGGCACATG
 - E=5e-66
CGCCCATGCCTGGCAAATTTTTGTATATTTAGTAGAGATGGGATTTAGC
```
